# Supplementary material for: The association of muscle weakness with functional disability in older patients with Diabetes mellitus: Measured by three different grip strength thresholds
Source: PLoS One. 2025 Jan 30;20(1):e0317250. doi: 10.1371/journal.pone.0317250 (PMC11781639; doi:10.1371/journal.pone.0317250)
Supplement: S1 Appendix — (DOCX) [file pone.0317250.s001.docx]

**S1 Appendix**

Appendix Table 1a. p values of the comparison between the AUCs for thresholds for ADLs in the DM group

|  | EWGSOP2 | Regional 32/22 kg | Regional 35/20 kg |
| --- | --- | --- | --- |
| EWGSOP2 | 1.000 | 0.19 | 0.20 |
| Regional 32/22 kg |  | 1.000 | 0.48 |
| Regional 35/20 kg |  |  | 1.000 |

Appendix Table 1b. z values of the comparison between the AUCs for thresholds for ADLs in the DM group

|  | Z value |
| --- | --- |
| EWGSOP2 vs. regional 32/22 kg | 0.87 |
| EWGSOP2 vs. regional 35/20 kg | 0.83 |
| Regional 32/22 kg vs. regional 35/20 kg | 0.04 |

Appendix Table 2a. p values of the comparison between the AUCs for thresholds for IADLs in the DM group

|  | EWGSOP2 | Regional 32/22 kg | Regional 35/20 kg |
| --- | --- | --- | --- |
| EWGSOP2 | 1.000 | 0.12 | 0.17 |
| Regional 32/22 kg |  | 1.000 | 0.42 |
| Regional 35/20 kg |  |  | 1.000 |

Appendix Table 2b. z values of the comparison between the AUCs for thresholds for IADLs in the DM group

|  | Z value |
| --- | --- |
| EWGSOP2 vs regional 32/22 kg | 1.16 |
| EWGSOP2 vs regional 35/20 kg | 0.97 |
| Regional 32/22 kg vs regional 35/20 kg | 0.19 |

Appendix Table 3a. p values of the comparison between the AUCs for thresholds for ADLs in the non-DM group

|  | EWGSOP2 | Regional 32/22 kg | Regional 35/20 kg |
| --- | --- | --- | --- |
| EWGSOP2 | 1.000 | 0.17 | 0.27 |
| Regional 32/22 kg |  | 1.000 | 0.36 |
| Regional 35/20 kg |  |  | 1.000 |

Appendix Table 3b. z values of the comparison between the AUCs for thresholds for ADLs in the non-DM group

|  | Z value |
| --- | --- |
| EWGSOP2 vs regional 32/22 kg | 0.97 |
| EWGSOP2 vs regional 35/20 kg | 0.62 |
| Regional 32/22 kg vs regional 35/20 kg | 0.37 |

Appendix Table 4a. p values of the comparison between the AUCs for thresholds for IADLs in the non-DM group

|  | EWGSOP2 | Regional 32/22 kg | Regional 35/20 kg |
| --- | --- | --- | --- |
| EWGSOP2 | 1.000 | 0.23 | 0.28 |
| Regional 32/22 kg |  | 1.000 | 0.43 |
| Regional 35/20 kg |  |  | 1.000 |

Appendix Table 4b. z values of the comparison between the AUCs for thresholds for IADLs in the non-DM group

|  | Z value |
| --- | --- |
| EWGSOP2 vs regional 32/22 kg | 0.74 |
| EWGSOP2 vs regional 35/20 kg | 0.57 |
| Regional 32/22 kg vs regional 35/20 kg | 0.18 |
